# Navigating the AI revolution: will radiology sink or soar?

**DOI:** 10.1007/s11604-025-01810-9

**Published:** 2025-07-31

**Authors:** Heinz-Peter Schlemmer

**Affiliations:** Department of Radiology, German Cancer Research Center, Im Neuenheimer Feld 280, 69120 Heidelberg, Germany

**Keywords:** Radiology, Medical Imaging, Digitalization, Artificial Intelligence, History

## Abstract

The rapid acceleration of digital transformation and artificial intelligence (AI) is fundamentally reshaping medicine. Much like previous technological revolutions, AI—driven by advances in computer technology and software including machine learning, computer vision, and generative models—is redefining cognitive work in healthcare. Radiology, as one of the first fully digitized medical specialties, is at the forefront of this transformation. AI is automating workflows, enhancing image acquisition and interpretation, and improving diagnostic precision, which collectively boost efficiency, reduce costs, and elevate patient care. Global data networks and AI-powered platforms are enabling borderless collaboration, empowering radiologists to focus on complex decision-making and patient interaction. Despite these profound opportunities, widespread AI adoption in radiology remains limited, often confined to specific use cases, such as chest, neuro, and musculoskeletal imaging. Concerns persist regarding transparency, explainability, and the ethical use of AI systems, while unresolved questions about workload, liability, and reimbursement present additional hurdles. Psychological and cultural barriers, including fears of job displacement and diminished professional autonomy, also slow acceptance. However, history shows that disruptive innovations often encounter initial resistance. Just as the discovery of X-rays over a century ago ushered in a new era, today, digitalization and artificial intelligence will drive another paradigm shift—this time through cognitive automation. To realize AI’s full potential, radiologists must maintain clinical oversight and safeguard their professional identity, viewing AI as a supportive tool rather than a threat. Embracing AI will allow radiologists to elevate their profession, enhance interdisciplinary collaboration, and help shape the future of medicine. Achieving this vision requires not only technological readiness but also early integration of AI education into medical training. Ultimately, radiology will not be replaced by AI, but by radiologists who effectively harness its capabilities.

Our journey into the digital future is accelerating at a dizzying pace. For the past 50 years, Moore’s Law has held true, with the number of transistors on a microchip doubling approximately every 2 years. The exponential growth in microprocessor performance has paved the way for groundbreaking evolution of computers [1]. Electronic computing turned now into reality what was once considered unimaginable—or even permanently out of reach. Since the dawn of the twenty-first century, computers have been able to replicate human cognition with ever-increasing precision—and in some areas, even to surpass it [2, 3]. The term "Artificial Intelligence" (AI) seamlessly found its way into everyday language, fueled by enthusiasm that goes beyond mere hype. Pandora’s box has been opened.

The digital revolution of the twenty-first century is reshaping cognitive work in much the same way the industrial revolution of the nineteenth century transformed physical labor. Cognitive work involves tasks traditionally requiring human intellect, such as decision-making, logistics optimization, medical image interpretation, and report writing. These tasks are increasingly automated by machines, a process known as cognitive automatization. This shift mirrors the historic replacement of manual labor by machines, fundamentally transforming industries and work itself. The term artificial intelligence (AI) refers to a broad spectrum of technologies, including machine learning (ML), deep learning (DL), natural language processing (NLP), computer vision, robotics, expert systems, and generative AI [4].

By emulating elements of human cognition, these technologies are increasingly capable of performing tasks traditionally carried out by highly skilled and creative professionals. Within a remarkably short period, rapid developments in computational technologies have begun to permeate virtually all aspects of life—transforming industries, reshaping societal structures, and influencing personal experiences. With technological advances accelerating at unprecedented rates, there’s growing concern that human cognition may struggle to keep pace and that these systems could spiral out of control—fueling fears that they might ultimately come to dominate our lives.

A study by the International Monetary Fund estimates that AI will impact 40% of global employment and 60% of jobs in advanced economies [5]. According to reports from the OECD (Organisation for Economic Co-operation and Development), professions requiring a university degree will be among the most affected, with individuals in higher-income groups being even more vulnerable than those in lower-income brackets [6, 7]. Affected are those professional groups that primarily engage in cognitive work in, e.g., administrative employees, legal professionals, tax specialists, data scientists, and even medical doctors and researchers. To escape the AI dominance, however, it will not be sufficient to just rely on traditional concepts and merely pursue higher education. Particularly advanced qualifications are at risk of automation by AI.

Great hope emerges, however, as digitalization, AI, and robotics offer promising opportunities to the pressing challenges facing healthcare systems worldwide, which are in urgent need of transformation. Population growth and increasing life expectancy, rapid urbanization, and the growing impact of adverse environmental and lifestyle factors—alongside rising costs of advanced diagnostics and treatment, and shortages of both financial and human resources—pose immense challenges.

AI presents significant opportunities to enhance the quality and efficiency of healthcare delivery [8]. Its ability to improve and automate both medical procedures and complex cognitive tasks within communication networks opens the door to a more equitable, accessible, and high-quality healthcare system, where healthcare professionals and scientists collaborate seamlessly within an integrated healthcare ecosystem.

It’s no surprise that the AI market in medical imaging is expanding rapidly, with projections estimating growth from $7.52 billion in 2025 to as much as $26.16 billion by 2030—driven largely by the fastest expansion in the Asia–Pacific region [9]. Key applications can be found not only in radiology but also in many other medical specialties dealing with imaging, including, e.g., in dermatology [10], ophthalmology [11] and cardiology [12]. Pathology is currently undergoing a profound transformation into full digitalization and AI-assisted diagnosis [13].

AI in radiology extends far beyond automated detection of image abnormalities and their medical interpretation. Radiology departments are rapidly evolving into large-scale, high-tech, high-throughput environments, with workloads growing continuously in both volume and complexity. Cognitive automation is set to transform every stage of the workflow—from order entry and scheduling to scanner selection, imaging-protocol optimization, worklist management, diagnostic support, report standardization, and seamless communication. By improving quality and safety while also reducing exam times and radiation exposure, AI can make radiology services markedly more efficient and effective [14–16].

For now, however, there remains a gap between the anticipated and the actual impact of AI on clinical practice. Currently, only around 30% of radiologists use AI into their routine clinical workflows [17]. Its use is largely limited to specific tasks—primarily the automated detection of nodules and pulmonary embolisms in chest CT, vessel occlusions and intracranial hemorrhages in neuro CT, as well as fracture detection, bone age estimation, and automated extremity measurements in musculoskeletal radiography [18].

Safety and ethical concerns of AI in clinical routine continue to be raised [19, 20]. Current generative AI and large multimodal foundation models goes beyond image interpretation and open windows to new opportunities for improving clinical workflows. A major concern is the lack of transparency of AI algorithms, which operate as 'black boxes'—prone to unknown biases and lacking explainability and reliability. AI systems may even generate so-called 'hallucinations', outputs that appear plausible but are, in fact, completely incorrect lacking any medical or scientific basis. Radiologists must carefully strike a balance between embracing innovation and mitigating potential risks [21]

In principle, AI applications could free up time for radiologists to enhance multidisciplinary communication and strengthen doctor–patient relationships. However, it is not far-fetched to consider that the freed-up time may be filled with new tasks, potentially leading to an even greater workload. In addition, as Radiologists will bear the ultimate responsibility for their patients, AI results will still require double checking which may further increase workload and even further intensify the feeling of being overwhelmed. Furthermore, reimbursement for AI applications remains a matter of ongoing debate. Clear evidence of clinical utility—demonstrating improved outcomes from both patient and societal perspectives—is needed to determine which AI tools merit additional compensation [22]. Accordingly, it remains a subject of debate whether AI may reduce burnout and improve job satisfaction or have rather the opposite effect [23].

In addition, radiologists may experience a deeper sense of internal resistance, driven by anxieties about their professional identity and job security. As AI systems advance and take over their tasks they may fear losing recognition for their expertise. Will Radiologists be relegated to the role of mere operators in automated imaging centers, eventually supplanted by AI-powered systems and robotic technologies?

But the transformation of radiology has already started and the AI market is expanding at an accelerating rate. Its impact on clinical practice, research, and education are expected to be extensive and profound. While some concerns may be valid, they should not stop Radiologists from harnessing the potential of AI and actively participating in shaping their role in the future. AI presents enormous opportunities with positive impacts on medicine. Concerns should rather play a constructive role in shaping progress and mitigating risks. In China AI is already widely recognized by radiology residents with 95.77% of being familiar with AI, machine learning, or big data analysis. The majority of radiologists hold an optimistic view of AI, with 72.80% believing it will enhance diagnostic accuracy and reduce errors. 78.18% feel that radiologists should actively embrace AI [17].

To embrace the ongoing transformation of radiology, one must look to the past, recognizing that today’s advancements are built upon the transformative breakthroughs of earlier times—some of which may have unsettled or even alarmed previous generations. Yet, with the benefit of hindsight, no one sought to oppose their emergence. The digital revolution of the twenty-first century, marked by the rise of cognitive automation through AI, is far from the first major transformation in the history of medicine. It is another pivotal moment in the enduring legacy of change, heralding a new era in which medicine will undergo further profound shifts Fig. 1.

**Fig. 1:**
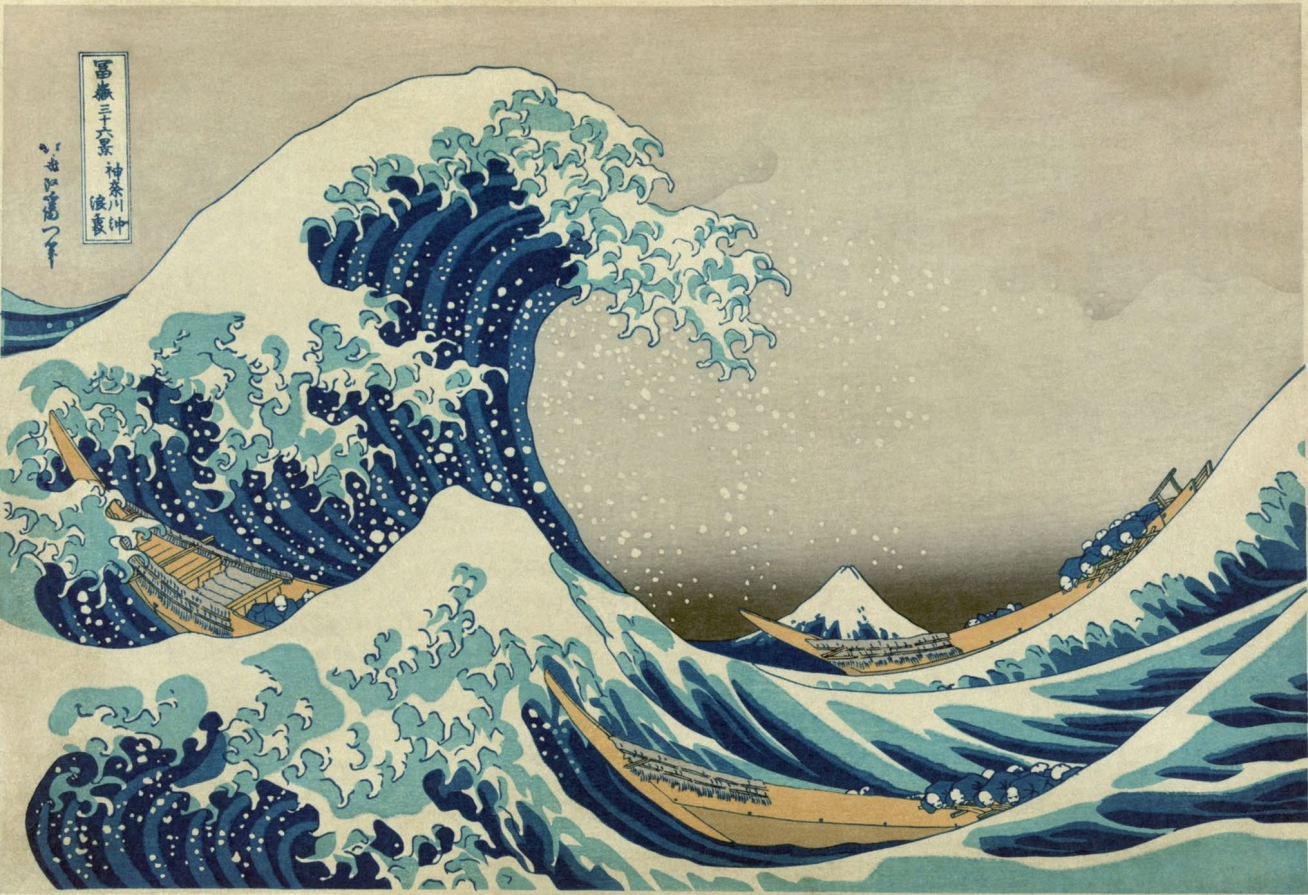
Great wave off Kanagawa, Katsushika Hokusai, 1830–1832

## A historical perspective on radiology

Radiology finds its roots in the discovery of X-rays in 1895, a moment that profoundly reshaped the scientific landscape. In medicine, it opened new frontiers for diagnosis and treatment, forever altering the course of healthcare. However, Wilhelm Conrad Röntgen’s groundbreaking discovery was made possible only by a series of earlier disruptive innovations, many of which arose during the Scientific and Industrial Revolutions that spanned the sixteenth to twentieth centuries. Notably, Michael Faraday’s research on electricity and magnetism and particularly his pivotal discovery of electromagnetic induction in 1831 was transformative [24]. His work laid the foundation for the development of electrical technologies, including generators, motors, and telegraphy, ultimately paving the way for modern radiological devices and electrical computing systems.

This was made possible by another pivotal advancement in technological history, the invention of steam engines and in addition their integration with electricity generators. This innovation enabled the consistent generation of electricity year-round, independent of the seasonal fluctuations of water and wind power. The economic potential of this breakthrough was quickly recognized. Large-scale power generation became a disruptive force, enabling the widespread electrification of industries, homes, and public spaces. This shift in energy production played a central role in accelerating industrial growth and fostering further technological innovation.

One technological keystone application of electricity was the generation of artificial light. The invention of the first practical incandescent light bulb by Thomas Edison in 1879 marked another key milestone in the evolution of artificial lighting technologies [25]. This breakthrough held immense market potential and catalyzed the pursuit of increasingly efficient technologies designed to produce high luminosity while minimizing heat loss.

During Röntgen's time, the study of visual luminescence phenomena in gas discharge tubes—also known as cathode ray tubes—was at the forefront of scientific research. These tubes were glass vessels of various designs, filled with gas and evacuated to different degrees. They were often named after their inventors, such as the Geissler tube, Hittorf tube, Crookes tube, and Lenard tube. While their construction and operation varied, all these tubes operated on the same fundamental principle: passing an electric current through a rarefied gas to produce visible effects. Like many of his contemporaries, Wilhelm Conrad Röntgen conducted his research using one of these gas discharge tubes. In addition, by doing this very carefully, he unexpectedly discovered in 1895 the by him named “X-rays”. Röntgen’s experiments with such a tube marked a disruptive breakthrough, evident in attracting immediate worldwide attention and earning him the very first Nobel Prize in Physics in 1901. Physicians, in particular, swiftly recognized its immense potential and rapidly incorporated it into both diagnostic and therapeutic practices. The rays revolutionized medical diagnostics and paved the way for further disruptive advancements in medical imaging [26].

The huge interest in X-rays within the medical community can be traced to a paradigm shift in medicine even back in the sixteenth century. In 1543, pioneering anatomist Andreas Vesalius published his groundbreaking work “De Humani Corporis Fabrica” (1543), revolutionizing the study of human anatomy and redefining medical science. He shifted the focus of the cause of diseases to anatomical abnormalities, away from the ancient Hippocratic theory of the four humors. In addition, Vesalius emphasized personal visual observation and dissection unlike his predecessors, who relied solely on ancient texts. His work led to a fundamentally new understanding of disease as anatomic anomalies. 350 years later this concept was revisited with the discovery of X-rays enabling non-invasive studying the human anatomy. The success of X-ray imaging stemmed from its unprecedented ability to visualize the inside of the human body—a feat previously possible only through autopsy. It was a disruptive breakthrough that anatomical abnormalities could from then on be observed noninvasively.

Initially, X-ray imaging was entirely analog. Röntgen used photographic films to document his X-ray images. This was possible only because of another groundbreaking development of the nineteenth century, the method of photography. It was a stroke of luck that Röntgen had a deep private dedication to photography and advanced plates with longer storage capacity were available to Röntgen at the time of his discovery [26].

In addition to exploring the potential of “photographic” imaging for medicine, radiologists were pioneers in digitalization and the application of computer technology in healthcare for over 50 years. From the 1970s, imaging with X-rays started to be digital. It was with the advent of electronic computers in the 1950s, that British engineer Godfrey Hounsfield developed computed tomography (CT), a groundbreaking technology that used electronic X-ray detectors and computer algorithms for image reconstruction. Those images existed solely as electronic data, which marked a paradigm shift toward digital technology in medical imaging. To acquire cross-sectional images of the human body without superimposition revolutionized medical diagnostics and earned Hounsfield the Nobel Prize in Physiology or Medicine 1979 and a knighthood in 1981. Pioneering work on digital flat panel detectors for X-rays started in the early 1990s, approximately at the same time as the development of digital sensors for visual photography started. Digital flat-panel X-ray detectors essentially correspond to those CCD detectors for conventional visual photography, even though different detector materials are required due to the different wavelengths of electromagnetic radiation of X-rays and light.

The digitization of medical imaging was a disruptive force that transformed radiology. It paved the way for other digital cross-sectional imaging techniques, including ultrasound (US), magnetic resonance imaging (MRI), single-photon emission computed tomography (SPECT), and positron emission tomography (PET). With these advancements, medical imaging became the first medical discipline to undergo full digitalization. The benefits of transitioning from analog to digital imaging were immediately apparent, opening up numerous opportunities: instant image visualization without the need for chemical processes, efficient storage, retrieval, sharing and management of vast data sets, virtually limitless post-processing options to improve image quality, and to extract quantitative and functional information.

Advanced image evaluation techniques, such as texture analysis, 3D reformation, image subtraction, and 4D data analysis, became possible and are now standard practice. Radiology departments underwent a profound transformation, evolving into fully digital organizations. Picture Archiving and Communication Systems (PACS), Radiology Information Systems (RIS), and digital speech recognition—all integrated within complex computer networks—were initially confined to radiology departments but eventually extended throughout the entire hospital, facilitating the rise of teleradiology services.

AI technologies are now being built on top of digital Radiology and will perform complex cognitive functions. The whole Radiology service will be improved and transformed. Global digital networking will extend these capabilities even further, enabling limitless exchange of radiology services across boarders. In addition, the ability to manage and transmit vast data sets over the internet—both locally and globally—will accelerate further advancements in AI, particularly those reliant on big data. Just as the discovery of X-rays 150 years ago revolutionized medicine and gave rise to the field of radiology, medicine today—led once again by radiology—is undergoing another profound transformation, this time driven by a powerful and disruptive force: artificial intelligence.

## Conclusion

Throughout history, disruptive technologies have driven medical progress, and the twenty-first-century digital revolution represents another significant leap forward. To stay ahead in the rapidly evolving digital transformation in radiology, strategic foresight and proactive collaboration are essential. The market for AI-driven solutions is expanding quickly, with many certified products expected to enter clinical practice, benefiting both patients and physicians. The joint involvement of medical professionals, policymakers, health insurers, pharmaceutical companies, industry associations, and research institutions is critical in shaping the integration of AI into healthcare.

Just as X-rays revolutionized medicine over 150 years ago, AI will elevate medical imaging and radiology to new heights, reshaping the profession and driving unprecedented advancements in patient care. The rapid expansion of the AI market underscores its impending impact. The future of radiology is not one of obsolescence but of immense opportunity, where AI serves as a powerful tool to enhance medical practice and patient outcomes. Radiologists will seize the opportunities to integrate AI into their practice.

AI’s ability to automate complex cognitive tasks is profoundly transformative. It will streamline scheduling, improve the quality of individual examinations, analyze increasingly large and complex multimodal imaging data sets, generate standardized diagnostic reports, and much more. It will optimize workflows not only within radiology departments but also across entire hospitals, enhancing communication among multidisciplinary teams and healthcare providers. As digital networking continues to evolve, radiology will become increasingly global, facilitating the seamless exchange of images and reports across borders. By taking over time-consuming tasks, AI will empower radiologists to devote more time to medical decision-making, interdisciplinary collaboration, and nurturing the doctor–patient relationship.

As AI continues to progress, it will not only augment the capabilities of radiologists but also transform their roles. By handling automatable tasks, AI will free radiologists to focus on areas requiring distinctly human expertise—such as overseeing complex systems, navigating intricate communication networks, and making high-stakes clinical decisions. In the future, their role may resemble that of airline pilots: operating sophisticated technology within highly computerized environments, while coordinating with remote experts through advanced communication systems. This evolution will enable radiologists to provide more efficient, accurate care, all while maintaining oversight and control over increasingly complex operations.

To prepare the next generation of radiologists for the challenges ahead, integrating AI into medical education from an early stage is essential. Future radiologists must be equipped not only with the knowledge and skills to leverage AI effectively but also to contribute to its development—while always maintaining their responsibility to patients. The benefits of AI in medicine are undeniable. Although initial skepticism may persist, it is likely to diminish as the technology’s transformative impact becomes increasingly evident. In fact, it may 1 day be considered unethical to perform high-responsibility medical tasks without the support of AI systems. By embracing AI responsibly and focusing on the human aspects of care, radiologists can fully unlock its potential, helping the field of radiology to continue to thrive.
